# [Se(CH_2_C(O)CH_3_)_3_][B_12_F_11_NH_3_]: The first selenium cation with three β-ketone substituents

**DOI:** 10.1107/S2056989020000481

**Published:** 2020-01-17

**Authors:** Carsten Jenne, Marc C. Nierstenhöfer

**Affiliations:** a Anorganische Chemie, Fakultät für Mathematik und Naturwissenschaften, Bergische Universität Wuppertal, Gaussstr. 20, 42119 Wuppertal, Germany

**Keywords:** crystal structure, selenium cation, weakly coordinating anion, dodeca­borates, boron cluster, C—H activation

## Abstract

The reaction of [Se_8_][B_12_F_11_NH_3_]_2_ with acetone and subsequent crystallization from acetone/diethyl ether yielded the selenium cation [Se(CH_2_C(O)CH_3_)_3_]^+^ as a by-product, which is stabilized by the weakly coordinating undeca­fluorinated anion [B_12_F_11_NH_3_]^−^. While attempting to crystallize pure [Se_8_][B_12_F_11_NH_3_]_2_, the structure of the isolated product, namely, tris­(2-oxoprop­yl)selenium 1-ammonio­undeca­fluoro­dodeca­borate, was surprising. The cation [Se(CH_2_C(O)CH_3_)_3_]^+^ represents the first example for a cationic selenium compound with three ketone functional groups located in the β-position with respect to the selenium atom. The cation possesses almost trigonal–pyramidal *C*_3_ symmetry and forms hydrogen bonds to the ammonio group of the anion.

## Abstract 2

The cation [Se(CH_2_C(O)CH_3_)_3_]^+^ represents the first example for a cationic selenium compound with three ketone functional groups located in the β-position with respect to the selenium atom. The cation possesses almost trigonal–pyramidal *C*_3_ symmetry and forms hydrogen bonds to the ammonio group of the anion

## Chemical context   

Homopolyatomic chalcogen cations are of fundamental importance in main-group chemistry because of their unusual structures and bonding situations (Brownridge *et al.*, 2000 ▸). Only a few examples for homopolyatomic selenium cations are known today, *e.g*. [Se_4_]^2+^ (Minkwitz *et al.*, 1991 ▸), [Se_8_]^2+^ (McMullan *et al.*, 1969 ▸), [Se_10_]^2+^ (Beck & Hilbert, 2000 ▸) and [Se_17_]^2+^ (Beck & Wetterau, 1995 ▸). These chalcogen cations are all dicationic and are all stabilized in the solid state by small perfluorinated or perchlorinated complex anions such as [SbF_6_]^−^ (Minkwitz *et al.*, 1991 ▸), [ReCl_6_]^2−^ (Beck *et al.*, 2002 ▸), or [AlCl_4_]^−^ (McMullan *et al.*, 1969 ▸). Recently, we were able to isolate the first homopolyatomic chalcogen radical cation, the sulfur cation [S_8_]^+^, and to determine its crystal structure (Derendorf *et al.*, 2017 ▸). The [S_8_]^+^ cation was stabilized in the solid state by the chlorinated *closo-*dodeca­borate anion [B_12_Cl_12_]^2−^. Consequently, the experimentally and theoretic­ally unknown corresponding radical cations of the chalcogen elements sulfur, selenium, and tellurium [*Ch_x_*]^+^ (*Ch* = S, Se, Te, *x* = 2–10) became of inter­est. In a very recent theoretical account, we have shown that some radical cations of the heavier chalcogens selenium and tellurium should also be experimentally accessible in condensed phases (Jenne & Nierstenhöfer, 2020 ▸). Modern weakly coordinating anions such as perhalogenated *closo-*dodeca­borates (Knapp, 2013 ▸) are expected to be suitable counter-anions for homopolyatomic chalcogen radical cations in solution and the solid state. For this purpose, the synthesis of [Se_8_][B_12_F_11_NH_3_]_2_ was attempted by a salt metathesis reaction of Na[B_12_F_11_NH_3_] and [Se_8_][AsF_6_]_2_ in liquid sulfur dioxide. The salt [Se_8_][B_12_F_11_NH_3_]_2_ was assumed to be a suitable precursor on the way to related open-shell radical cations. From an attempt to generate single crystals of this compound from acetone/diethyl ether solution, the organoselenium cation [Se(CH_2_C(O)CH_3_)_3_]^+^ was isolated as the [B_12_F_11_NH_3_]^−^ salt.
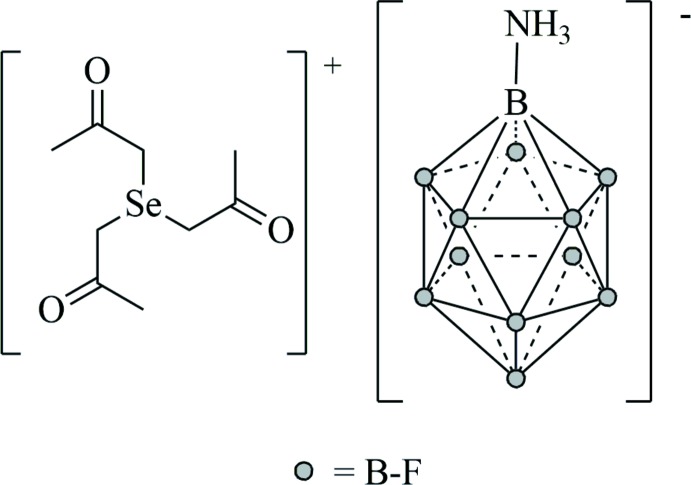


Homopolyatomic selenium cations have not been considered before for addition or substitution reactions on ketones or enols. However, the reaction of homopolyatomic sulfur cations with aceto­nitrile under C—H activation has been reported (Cameron *et al.*, 1999 ▸). Typically, alkyl selenium halides (*R*Se*X*) such as C_6_H_5_SeAl*R*_2_ (Reich *et al.*, 1975 ▸), C_5_H_4_NSeCl (Kozikowski & Ames, 1978 ▸), or dialkyl selenium compounds *R*_2_Se such as (C_6_H_5_)_2_Se_2_ (Toshimitsu *et al.*, 1984 ▸) have been used for electrophilic selenylation of functionalized olefins or enolisable ketones such as acetone. After a subsequent oxidation, α,β-unsaturated or 1,2 diketones can be obtained (Reich *et al.*, 1979 ▸; Marshall & Royce 1982 ▸; Schreiber & Santini, 1984 ▸). Organoselenium cations are well known. The most simple representatives are trialkyl- or triaryl-substituted cations such as [Me_3_Se]^+^ (Hope, 1966 ▸) and [Ph_3_Se]^+^ (Leicester & Bergstrom, 1929 ▸), but mixed derivatives such as [Ph_2_MeSe]^+^ (Dumont *et al.*, 1974 ▸) are known as well. Furthermore, selenium cations with a single keto group {[PhC(O)CH_2_SeMe_2_]^+^; Lotz & Gosselck, 1973 ▸} or a carbonic acid {[Me_2_SeCH_2_COOH]^+^; Ip & Ganther, 1990 ▸} in the *β*-position have been reported. A symmetrically substituted selenium cation with three *β*-keto groups is reported herein for the first time.

## Structural commentary   

**Figure 1:**
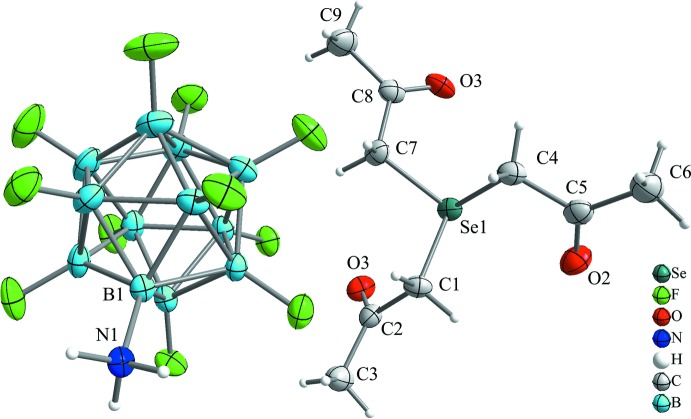
Part of the crystal structure of [Se(CH_2_C(O)CH_3_)_3_][B_12_F_11_NH_3_]. Displacement ellipsoids are drawn at the 50% probability level and hydrogen atoms are shown with arbitrary radii.

The salt [Se(CH_2_C(O)CH_3_)_3_][B_12_F_11_NH_3_] crystallizes solvent-free in the ortho­rhom­bic crystal system in space group *Pbca* (Fig. 1 ▸). The cation is close to being *C*_3_ symmetric and the selenium atom is bonded to three chemically equivalent methyl­ene groups with essentially identical bond lengths of 1.947 (2) to 1.951 (2) Å (Table 1 ▸). These distances are in the expected range when compared to the simple [SeMe_3_]^+^ cation (Hope, 1966 ▸) with Se—C bond lengths from 1.94 (2) to 1.96 (2) Å. This indicates that the electron-withdrawing effect of the ketone groups has no influence on the Se—C bond lengths.

**Figure 2:**
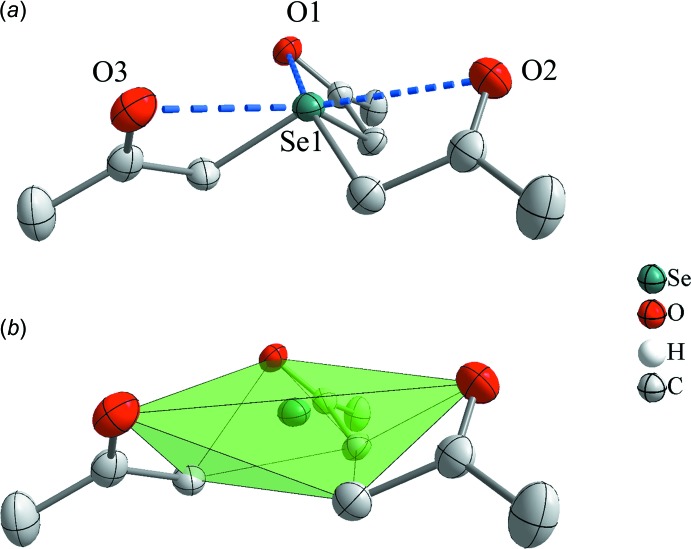
(*a*) The [Se(CH_2_C(O)CH_3_)_3_]^+^ cation shows intra­molecular contacts of the selenium atom to the three oxygen atoms of the ketone groups. Selected intra­molecular contacts are drawn using dashed lines [O1⋯Se1 = 2.794 (3), O2⋯Se1 = 2.788 (3) and O3—Se1 = 2.847 (3) Å]. (*b*) Coordination sphere around the Se atom forming a distorted trigonal prism. Displacement ellipsoids are drawn at the 50% probability level and hydrogen atoms are omitted for clarity.

Furthermore, there are additional contacts between the central selenium cation and the three oxygen atoms of the ketone groups (Fig. 2 ▸ *a*). The oxygen–selenium contacts (2.810 Å on average) are much shorter than the sum of the van der Waals radii of selenium and oxygen (3.42 Å; Bondi, 1964 ▸). This inter­action can be considered as mainly electrostatic, since the oxygen atoms are partially negatively charged and the selenium cation carries a positive charge. Thus, the selenium atom forms six short contacts, *i.e.* it is covalently bonded to three carbon atoms and forms three ionic inter­actions to the three oxygen atoms. The carbon atoms span a small triangular face, which is essentially parallel to a larger triangular face formed by the oxygen atoms (Fig. 2 ▸ *b*). This results in a flat distorted trigonal prism surrounding the selenium atom.

The structure of the anion [B_12_F_11_NH_3_]^−^ is less inter­esting and reveals bond distances in the expected range. The boron–boron bond lengths in the anion are in the range 1.777 (3) to 1.803 (4) Å and the average boron–fluorine bond length is 1.38 Å, which are very similar to those in other fluorinated dodeca­borates such as [B_12_F_11_NMe_3_]^−^ (Strauss *et al.*, 2003 ▸) or [B_12_F_12_]^2−^ (Ivanov *et al.*, 2003 ▸). The B—N bond length of 1.538 (3) Å is essentially equal to that in [B_12_H_11_NH_3_]^−^ (Nachtigal *et al.*, 1997 ▸) but slightly shorter than in [B_12_F_11_NMe_3_]^−^ (Strauss *et al.*, 2003 ▸) and in [B_12_Cl_11_NMe_3_]^−^ (Bolli *et al.*, 2014 ▸).

## Supra­molecular features   

**Figure 3:**
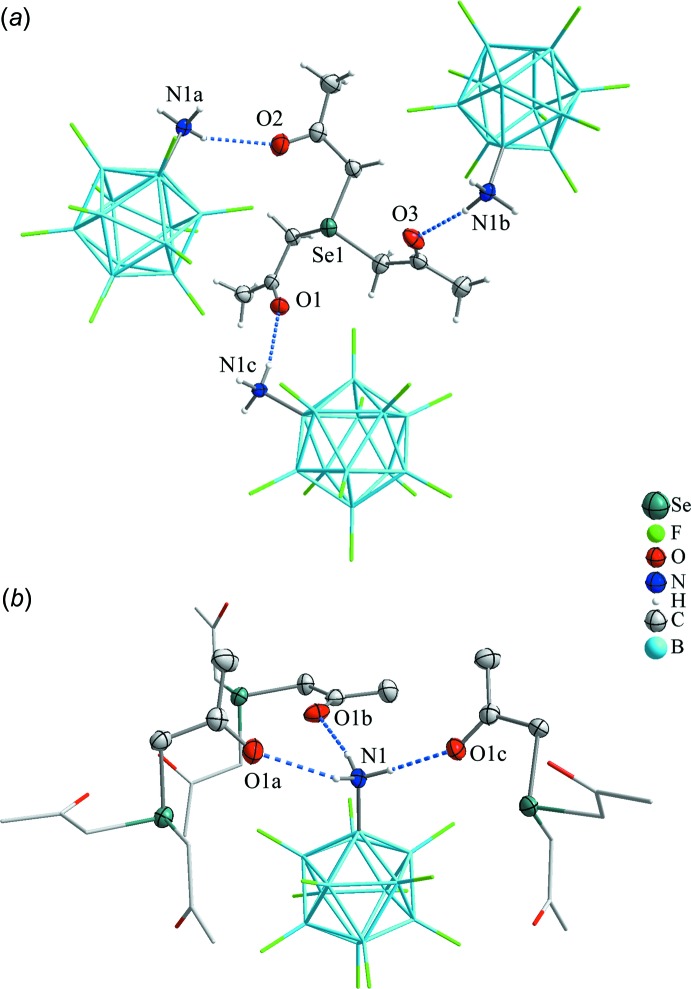
Inter­molecular hydrogen-bonding inter­actions (dashed lines) between the hydrogen atoms of the ammonio group of the boron cluster anions and the oxygen atoms of the ketone functions. Displacement ellipsoids are drawn at the 50% probability level and hydrogen atoms are shown with arbitrary radii. Symmetry codes: (*a*) *x*, 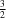 − *y*, *z*; (*b*) 1 − *x*, −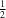 + *y*, 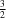 − *z*; (*c*) −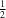 + *x*, *y*, 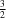 − *z*.

The [Se(CH_2_C(O)CH_3_)_3_]^+^ cations and the [B_12_F_11_NH_3_]^−^ anions are connected by inter­molecular hydrogen-bonding inter­actions, resulting in a polymeric network. The hydrogen atoms of the positively charged ammonio group of the cation inter­act with the partially negatively charged oxygen atoms of the ketone groups of the cation (Fig. 3 ▸ *a*). The oxygen–nitro­gen distances are between 2.841 (2) and 2.865 (2) Å (Table 2 ▸), which is in the range of typical N—H⋯O hydrogen bonds (Huheey, 1988 ▸). Every ketone group of the cation is coordinated to an ammonio group of a different boron cluster anion. Likewise every ammonio group is coordinated threefold by the ketone groups of three different cations, as shown in Fig. 3 ▸ *b*.

## Database survey   

The weakly coordinating anion [B_12_F_11_NH_3_]^−^ was first reported in 2003 and was further functionalized by methyl­ation yielding the [B_12_F_11_NMe_3_]^−^ anion (Ivanov *et al.*, 2003 ▸). Only two crystal structures of the non-alkyl­ated ammonio-functionalized, undeca­fluoro dodeca­borate are known, *i.e.* the sodium tetra­aqua complex [Na(H_2_O)_4_]^+^ (Strauss *et al.*, 2017 ▸) and the solvent-free K[B_12_F_11_NH_3_] salt (Jenne & Nierstenhöfer, 2019 ▸). The crystal structures of simple organoselenium cations with three alkyl or aryl substituents, *e.g.* [Ph_2_MeSe]^+^ (Dumont *et al.*, 1974 ▸), [Me_3_Se]^+^ (Hope, 1966 ▸), or [Ph_3_Se]^+^ (Leicester & Bergstrom, 1929 ▸), and a selenium cation with one *β*-ketone substituent and two methyl groups {[Me_2_SeCH_2_COOH]^+^; Ip & Ganther, 1990 ▸} have been previously reported.

## Synthesis and crystallization   

[Se_8_][B_12_F_11_NH_3_]_2_ was prepared by a salt metathesis reaction of Na[B_12_F_11_NH_3_] with [Se_8_][AsF_6_]_2_ in liquid sulfur dioxide as a solvent in an H-shaped glass vessel with an incorporated frit. The insoluble by-product Na[AsF_6_] was removed by filtration. The soluble black residue was dissolved in acetone in order to obtain single crystals of the intended compound [Se_8_][B_12_F_11_NH_3_]_2_. The title compound was obtained as a by-product from the reaction of [Se_8_][B_12_F_11_NH_3_]_2_ with acetone. Single crystals were grown by slow diffusion of diethyl ether into a saturated solution of acetone at room temperature. During crystallization a red precipitate formed, which hints at the formation of elemental red selenium. We assume that the formation of the title cation is the result of a C—H activation of acetone by the [Se_8_]^2+^ cation.

## Refinement   

Crystal data, data collection and structure refinement details are summarized in Table 3 ▸. H atoms were positioned geometrically (N—H = 0.91 or C—H = 0.95–0.99 °) and refined using a riding model with *U*_iso_(H) = 1.2*U*_eq_(C, N) or 1.5*U*_eq_(C-meth­yl).

## Supplementary Material

Crystal structure: contains datablock(s) I. DOI: 10.1107/S2056989020000481/pk2622sup1.cif

Structure factors: contains datablock(s) I. DOI: 10.1107/S2056989020000481/pk2622Isup2.hkl

CCDC reference: 1977644

Additional supporting information:  crystallographic information; 3D view; checkCIF report

## Figures and Tables

**Table 1:** Selected geometric parameters (Å, °)

Se1—C1	1.9511 (19)	O2—C5	1.211 (3)
Se1—C4	1.951 (2)	C2—C1	1.508 (3)
Se1—C7	1.947 (2)	C5—C4	1.509 (3)
O1—C2	1.212 (2)	C8—C7	1.507 (3)
O3—C8	1.205 (3)		
			
C4—Se1—C1	98.17 (9)	C7—Se1—C4	97.67 (9)
C7—Se1—C1	98.26 (9)		

**Table 2:** Hydrogen-bond geometry (Å, °)

*D*—H⋯*A*	*D*—H	H⋯*A*	*D*⋯*A*	*D*—H⋯*A*
N1—H⋯O2^i^	0.91	2.20	2.865 (2)	129
N1—H*A*⋯O1^ii^	0.91	1.94	2.836 (2)	168
N1—H*B*⋯O3^iii^	0.91	1.94	2.841 (2)	171

Symmetry codes: (i) 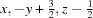; (ii) 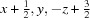; (iii) 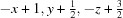.

**Table 3:** Experimental details

Crystal data	
Chemical formula	C_9_H_15_O_3_Se^+^·B_12_F_11_H_3_N^−^
*M*_r_	605.92
Crystal system, space group	Orthorhombic, *P* *b* *c* *a*
Temperature (K)	150
*a*, *b*, *c* (Å)	12.6157 (3), 16.9629 (4), 22.2759 (6)
*V* (Å^3^)	4767.0 (2)
*Z*	8
Radiation type	Mo *K*α
μ (mm^−1^)	1.68
Crystal size (mm)	0.17 × 0.11 × 0.06

Data collection	
Diffractometer	Rigaku Oxford Diffraction Xcalibur, Eos, Gemini ultra
Absorption correction	Multi-scan (*CrysAlis PRO*; Rigaku OD, 2015 ▸)
*T*_min_, *T*_max_	0.758, 1.000
No. of measured, independent and observed [*I* > 2σ(*I*)] reflections	17080, 5719, 4622
*R*_int_	0.024
(sin θ/λ)_max_ (Å^−1^)	0.693

Refinement	
*R*[*F* ^2^ > 2σ(*F* ^2^)], *wR*(*F* ^2^), *S*	0.032, 0.087, 1.03
No. of reflections	5719
No. of parameters	338
H-atom treatment	H-atom parameters constrained
Δρ_max_, Δρ_min_ (e Å^−3^)	0.45, −0.42

Computer programs: *CrysAlis PRO* (Rigaku OD, 2015 ▸), *SHELXT* (Sheldrick, 2015*a* ▸), *SHELXL* (Sheldrick, 2015*b* ▸) and *OLEX2* (Dolomanov *et al.*, 2009 ▸).

## supplementary crystallographic information

### Crystal data

**Table:**

C_9_H_15_O_3_Se^+^·B_12_F_11_H_3_N^−^	*D*_x_ = 1.689 Mg m^−^^3^
*M**_r_* = 605.92	Mo *K*α radiation, λ = 0.71073 Å
Orthorhombic, *P**b**c**a*	Cell parameters from 5874 reflections
*a* = 12.6157 (3) Å	θ = 3.0–29.2°
*b* = 16.9629 (4) Å	µ = 1.68 mm^−^^1^
*c* = 22.2759 (6) Å	*T* = 150 K
*V* = 4767.0 (2) Å^3^	Block, clear light colourless
*Z* = 8	0.17 × 0.11 × 0.06 mm
*F*(000) = 2368	

### Data collection

**Table:**

Rigaku Oxford Diffraction Xcalibur, Eos, Gemini ultra diffractometer	5719 independent reflections
Radiation source: fine-focus sealed X-ray tube, Enhance (Mo) X-ray Source	4622 reflections with *I* > 2σ(*I*)
Graphite monochromator	*R*_int_ = 0.024
Detector resolution: 16.2705 pixels mm^-1^	θ_max_ = 29.5°, θ_min_ = 2.7°
ω scans	*h* = −17→14
Absorption correction: multi-scan (CrysAlisPro; Rigaku OD, 2015)	*k* = −23→14
*T*_min_ = 0.758, *T*_max_ = 1.000	*l* = −30→26
17080 measured reflections	

### Refinement

**Table:**

Refinement on *F*^2^	Primary atom site location: structure-invariant direct methods
Least-squares matrix: full	Hydrogen site location: inferred from neighbouring sites
*R*[*F*^2^ > 2σ(*F*^2^)] = 0.032	H-atom parameters constrained
*wR*(*F*^2^) = 0.087	*w* = 1/[σ^2^(*F*_o_^2^) + (0.0441*P*)^2^ + 2.0302*P*] where *P* = (*F*_o_^2^ + 2*F*_c_^2^)/3
*S* = 1.03	(Δ/σ)_max_ = 0.002
5719 reflections	Δρ_max_ = 0.45 e Å^−^^3^
338 parameters	Δρ_min_ = −0.42 e Å^−^^3^
0 restraints	

### Special details

**Table:**

Geometry. All esds (except the esd in the dihedral angle between two l.s. planes) are estimated using the full covariance matrix. The cell esds are taken into account individually in the estimation of esds in distances, angles and torsion angles; correlations between esds in cell parameters are only used when they are defined by crystal symmetry. An approximate (isotropic) treatment of cell esds is used for estimating esds involving l.s. planes.

### Fractional atomic coordinates and isotropic or equivalent isotropic displacement parameters (Å^2^)

**Table:**

	*x*	*y*	*z*	*U*_iso_*/*U*_eq_	
Se1	0.48873 (2)	0.60776 (2)	0.89752 (2)	0.02430 (7)	
F11	0.42902 (10)	0.72825 (7)	0.75079 (5)	0.0307 (3)	
F5	0.67805 (10)	0.73550 (8)	0.71785 (6)	0.0336 (3)	
F6	0.51832 (10)	0.87887 (7)	0.68324 (6)	0.0313 (3)	
F2	0.46146 (10)	0.86422 (9)	0.54175 (6)	0.0383 (3)	
F3	0.58351 (11)	0.71123 (9)	0.48901 (6)	0.0407 (3)	
F4	0.71576 (10)	0.63101 (7)	0.59821 (6)	0.0373 (3)	
F10	0.54740 (11)	0.57225 (7)	0.69968 (7)	0.0402 (3)	
F12	0.31082 (10)	0.61473 (8)	0.65310 (7)	0.0424 (3)	
F7	0.29441 (9)	0.80596 (8)	0.64224 (6)	0.0414 (3)	
O1	0.40468 (11)	0.75890 (9)	0.88266 (7)	0.0324 (3)	
O3	0.34256 (12)	0.48099 (9)	0.88486 (8)	0.0347 (4)	
F8	0.33176 (11)	0.70261 (11)	0.52287 (7)	0.0569 (4)	
F9	0.48962 (12)	0.55469 (9)	0.55813 (8)	0.0560 (5)	
O2	0.65887 (13)	0.58953 (10)	0.97614 (7)	0.0402 (4)	
N1	0.70624 (12)	0.82307 (9)	0.58287 (7)	0.0203 (3)	
H	0.712266	0.822528	0.542164	0.024*	
HA	0.766087	0.802626	0.599535	0.024*	
HB	0.697272	0.873550	0.595723	0.024*	
C2	0.49612 (15)	0.76780 (13)	0.86736 (10)	0.0261 (4)	
C1	0.56931 (15)	0.69751 (11)	0.86722 (10)	0.0255 (4)	
H1A	0.631476	0.707672	0.893214	0.031*	
H1B	0.594761	0.686763	0.825987	0.031*	
C5	0.67037 (16)	0.53359 (13)	0.94298 (10)	0.0303 (5)	
C8	0.34198 (16)	0.51183 (12)	0.83614 (11)	0.0301 (5)	
C4	0.59842 (17)	0.52743 (12)	0.88906 (10)	0.0298 (5)	
H4A	0.565919	0.474365	0.887190	0.036*	
H4B	0.639274	0.536213	0.851705	0.036*	
C7	0.40988 (17)	0.58340 (13)	0.82470 (9)	0.0289 (4)	
H7A	0.364670	0.628733	0.813401	0.035*	
H7B	0.459606	0.572818	0.791277	0.035*	
C3	0.54099 (18)	0.84483 (13)	0.84772 (12)	0.0370 (5)	
H3A	0.483168	0.880480	0.836498	0.056*	
H3B	0.587517	0.836547	0.813056	0.056*	
H3C	0.581813	0.868127	0.880676	0.056*	
B1	0.61004 (16)	0.77319 (13)	0.60178 (10)	0.0197 (4)	
B5	0.60007 (17)	0.72730 (13)	0.67335 (10)	0.0218 (4)	
B7	0.38811 (18)	0.76623 (15)	0.63220 (11)	0.0273 (5)	
C6	0.75232 (19)	0.47173 (14)	0.95177 (14)	0.0460 (6)	
H6A	0.789968	0.462767	0.913900	0.069*	
H6B	0.718250	0.422654	0.964678	0.069*	
H6C	0.802785	0.488972	0.982531	0.069*	
C9	0.2774 (2)	0.48398 (15)	0.78432 (13)	0.0484 (7)	
H9A	0.247527	0.432053	0.793526	0.073*	
H9B	0.322503	0.480121	0.748599	0.073*	
H9C	0.219847	0.521441	0.776727	0.073*	
B11	0.46184 (17)	0.72269 (13)	0.69204 (10)	0.0229 (4)	
B4	0.62094 (18)	0.66877 (13)	0.60733 (11)	0.0248 (5)	
B3	0.54671 (19)	0.71304 (15)	0.54721 (11)	0.0287 (5)	
B6	0.51274 (16)	0.80691 (13)	0.65415 (10)	0.0210 (4)	
B8	0.40916 (19)	0.70841 (18)	0.56625 (11)	0.0346 (6)	
B12	0.39774 (18)	0.66108 (15)	0.63797 (11)	0.0297 (5)	
B10	0.52843 (18)	0.63720 (14)	0.66360 (12)	0.0266 (5)	
B2	0.48040 (17)	0.79836 (16)	0.57652 (10)	0.0265 (5)	
B9	0.4957 (2)	0.62763 (17)	0.58525 (13)	0.0345 (6)	

### Atomic displacement parameters (Å^2^)

**Table:**

	*U*^11^	*U*^22^	*U*^33^	*U*^12^	*U*^13^	*U*^23^
Se1	0.02537 (11)	0.02575 (12)	0.02179 (11)	−0.00251 (8)	0.00435 (8)	−0.00125 (8)
F11	0.0338 (6)	0.0364 (7)	0.0220 (6)	0.0001 (5)	0.0069 (5)	0.0056 (5)
F5	0.0289 (6)	0.0450 (7)	0.0269 (6)	0.0002 (6)	−0.0052 (5)	0.0055 (6)
F6	0.0354 (7)	0.0240 (6)	0.0344 (7)	0.0013 (5)	0.0103 (5)	−0.0022 (5)
F2	0.0287 (6)	0.0525 (8)	0.0335 (7)	0.0104 (6)	0.0019 (5)	0.0215 (6)
F3	0.0429 (7)	0.0585 (9)	0.0208 (6)	−0.0120 (7)	0.0056 (6)	−0.0084 (6)
F4	0.0304 (6)	0.0282 (6)	0.0533 (9)	0.0073 (5)	0.0105 (6)	−0.0017 (6)
F10	0.0412 (7)	0.0284 (6)	0.0509 (9)	0.0057 (6)	0.0125 (7)	0.0147 (6)
F12	0.0295 (7)	0.0503 (8)	0.0475 (8)	−0.0186 (6)	0.0048 (6)	−0.0008 (7)
F7	0.0183 (6)	0.0579 (9)	0.0480 (8)	0.0104 (6)	0.0064 (5)	0.0182 (7)
O1	0.0223 (7)	0.0366 (8)	0.0383 (9)	0.0016 (6)	0.0042 (6)	−0.0074 (7)
O3	0.0341 (8)	0.0224 (7)	0.0476 (10)	−0.0006 (6)	0.0103 (7)	0.0050 (7)
F8	0.0358 (7)	0.0992 (13)	0.0357 (8)	−0.0223 (8)	−0.0166 (6)	0.0007 (8)
F9	0.0596 (10)	0.0477 (9)	0.0608 (10)	−0.0261 (7)	0.0160 (8)	−0.0285 (8)
O2	0.0400 (9)	0.0458 (10)	0.0347 (9)	−0.0003 (8)	−0.0040 (7)	−0.0113 (8)
N1	0.0174 (7)	0.0226 (8)	0.0209 (8)	0.0032 (6)	0.0019 (6)	0.0011 (7)
C2	0.0240 (10)	0.0285 (10)	0.0258 (10)	0.0009 (8)	−0.0029 (8)	−0.0038 (8)
C1	0.0214 (9)	0.0239 (10)	0.0314 (11)	−0.0034 (8)	0.0055 (8)	−0.0025 (8)
C5	0.0267 (10)	0.0297 (11)	0.0345 (12)	−0.0060 (9)	0.0017 (9)	0.0017 (9)
C8	0.0243 (10)	0.0242 (10)	0.0417 (13)	0.0035 (8)	0.0008 (9)	−0.0022 (9)
C4	0.0314 (11)	0.0237 (10)	0.0343 (12)	−0.0015 (9)	0.0006 (9)	−0.0032 (9)
C7	0.0297 (11)	0.0330 (11)	0.0239 (10)	−0.0049 (9)	0.0012 (8)	0.0004 (9)
C3	0.0310 (11)	0.0283 (11)	0.0517 (15)	−0.0009 (10)	−0.0049 (10)	0.0026 (11)
B1	0.0159 (9)	0.0226 (10)	0.0204 (10)	0.0021 (8)	0.0009 (8)	0.0018 (8)
B5	0.0184 (10)	0.0265 (11)	0.0204 (10)	0.0018 (9)	−0.0026 (8)	0.0023 (9)
B7	0.0178 (10)	0.0385 (13)	0.0255 (12)	0.0011 (10)	−0.0007 (9)	0.0058 (10)
C6	0.0332 (12)	0.0346 (12)	0.0700 (19)	−0.0007 (10)	−0.0079 (12)	0.0005 (13)
C9	0.0381 (13)	0.0408 (13)	0.0664 (19)	−0.0030 (11)	−0.0167 (13)	−0.0051 (13)
B11	0.0203 (10)	0.0259 (11)	0.0226 (11)	0.0005 (9)	0.0025 (9)	0.0042 (9)
B4	0.0229 (10)	0.0217 (10)	0.0297 (12)	0.0000 (9)	0.0040 (9)	−0.0023 (9)
B3	0.0265 (11)	0.0383 (13)	0.0213 (11)	−0.0075 (10)	0.0007 (9)	−0.0052 (10)
B6	0.0186 (10)	0.0236 (10)	0.0207 (10)	0.0024 (8)	0.0020 (8)	0.0038 (9)
B8	0.0235 (11)	0.0546 (16)	0.0257 (12)	−0.0107 (11)	−0.0066 (10)	−0.0014 (11)
B12	0.0223 (11)	0.0358 (13)	0.0309 (13)	−0.0100 (10)	0.0012 (9)	0.0010 (11)
B10	0.0257 (11)	0.0228 (11)	0.0313 (12)	−0.0006 (9)	0.0039 (9)	0.0023 (10)
B2	0.0184 (10)	0.0408 (13)	0.0204 (11)	0.0017 (10)	−0.0011 (8)	0.0073 (10)
B9	0.0344 (13)	0.0347 (13)	0.0344 (13)	−0.0116 (11)	0.0052 (11)	−0.0099 (11)

### Geometric parameters (Å, º)

**Table:**

Se1—C1	1.9511 (19)	B1—B5	1.779 (3)
Se1—C4	1.951 (2)	B1—B4	1.781 (3)
Se1—C7	1.947 (2)	B1—B3	1.777 (3)
F11—B11	1.376 (3)	B1—B6	1.787 (3)
F5—B5	1.404 (2)	B1—B2	1.781 (3)
F6—B6	1.384 (3)	B5—B11	1.795 (3)
F2—B2	1.380 (3)	B5—B4	1.794 (3)
F3—B3	1.378 (3)	B5—B6	1.794 (3)
F4—B4	1.372 (3)	B5—B10	1.789 (3)
F10—B10	1.384 (3)	B7—B11	1.785 (3)
F12—B12	1.391 (3)	B7—B6	1.785 (3)
F7—B7	1.379 (3)	B7—B8	1.786 (4)
O1—C2	1.212 (2)	B7—B12	1.792 (4)
O3—C8	1.205 (3)	B7—B2	1.786 (3)
F8—B8	1.377 (3)	C6—H6A	0.9800
F9—B9	1.379 (3)	C6—H6B	0.9800
O2—C5	1.211 (3)	C6—H6C	0.9800
N1—H	0.9100	C9—H9A	0.9800
N1—HA	0.9100	C9—H9B	0.9800
N1—HB	0.9100	C9—H9C	0.9800
N1—B1	1.538 (3)	B11—B6	1.779 (3)
C2—C1	1.508 (3)	B11—B12	1.788 (3)
C2—C3	1.490 (3)	B11—B10	1.792 (3)
C1—H1A	0.9900	B4—B3	1.798 (4)
C1—H1B	0.9900	B4—B10	1.794 (3)
C5—C4	1.509 (3)	B4—B9	1.795 (3)
C5—C6	1.486 (3)	B3—B8	1.788 (3)
C8—C7	1.507 (3)	B3—B2	1.795 (4)
C8—C9	1.489 (3)	B3—B9	1.797 (4)
C4—H4A	0.9900	B6—B2	1.783 (3)
C4—H4B	0.9900	B8—B12	1.794 (4)
C7—H7A	0.9900	B8—B2	1.786 (4)
C7—H7B	0.9900	B8—B9	1.803 (4)
C3—H3A	0.9800	B12—B10	1.791 (3)
C3—H3B	0.9800	B12—B9	1.797 (4)
C3—H3C	0.9800	B10—B9	1.801 (4)
			
C4—Se1—C1	98.17 (9)	F4—B4—B10	122.08 (18)
C7—Se1—C1	98.26 (9)	F4—B4—B9	123.03 (18)
C7—Se1—C4	97.67 (9)	B1—B4—B5	59.67 (12)
H—N1—HA	109.5	B1—B4—B3	59.52 (13)
H—N1—HB	109.5	B1—B4—B10	107.16 (15)
HA—N1—HB	109.5	B1—B4—B9	107.43 (17)
B1—N1—H	109.5	B5—B4—B3	107.63 (16)
B1—N1—HA	109.5	B5—B4—B10	59.81 (13)
B1—N1—HB	109.5	B5—B4—B9	108.09 (16)
O1—C2—C1	118.99 (19)	B10—B4—B3	107.82 (16)
O1—C2—C3	123.6 (2)	B10—B4—B9	60.21 (14)
C3—C2—C1	117.40 (17)	B9—B4—B3	60.01 (15)
Se1—C1—H1A	110.3	F3—B3—B1	120.35 (18)
Se1—C1—H1B	110.3	F3—B3—B4	121.07 (19)
C2—C1—Se1	107.29 (13)	F3—B3—B8	123.31 (19)
C2—C1—H1A	110.3	F3—B3—B2	121.2 (2)
C2—C1—H1B	110.3	F3—B3—B9	123.1 (2)
H1A—C1—H1B	108.5	B1—B3—B4	59.75 (13)
O2—C5—C4	117.9 (2)	B1—B3—B8	107.42 (16)
O2—C5—C6	123.8 (2)	B1—B3—B2	59.84 (12)
C6—C5—C4	118.3 (2)	B1—B3—B9	107.53 (17)
O3—C8—C7	119.9 (2)	B8—B3—B4	108.09 (17)
O3—C8—C9	124.3 (2)	B8—B3—B2	59.79 (14)
C9—C8—C7	115.8 (2)	B8—B3—B9	60.37 (15)
Se1—C4—H4A	110.2	B2—B3—B4	107.98 (16)
Se1—C4—H4B	110.2	B2—B3—B9	108.17 (17)
C5—C4—Se1	107.53 (14)	B9—B3—B4	59.91 (14)
C5—C4—H4A	110.2	F6—B6—B1	123.57 (16)
C5—C4—H4B	110.2	F6—B6—B5	121.40 (17)
H4A—C4—H4B	108.5	F6—B6—B7	120.93 (16)
Se1—C7—H7A	110.0	F6—B6—B11	120.30 (17)
Se1—C7—H7B	110.0	F6—B6—B2	122.52 (17)
C8—C7—Se1	108.69 (15)	B1—B6—B5	59.55 (12)
C8—C7—H7A	110.0	B7—B6—B1	107.61 (16)
C8—C7—H7B	110.0	B7—B6—B5	108.37 (16)
H7A—C7—H7B	108.3	B11—B6—B1	107.49 (15)
C2—C3—H3A	109.5	B11—B6—B5	60.29 (12)
C2—C3—H3B	109.5	B11—B6—B7	60.11 (13)
C2—C3—H3C	109.5	B11—B6—B2	108.19 (16)
H3A—C3—H3B	109.5	B2—B6—B1	59.87 (12)
H3A—C3—H3C	109.5	B2—B6—B5	108.09 (16)
H3B—C3—H3C	109.5	B2—B6—B7	60.09 (13)
N1—B1—B5	122.82 (16)	F8—B8—B7	120.7 (2)
N1—B1—B4	120.34 (16)	F8—B8—B3	121.66 (19)
N1—B1—B3	118.90 (16)	F8—B8—B12	122.41 (19)
N1—B1—B6	123.00 (16)	F8—B8—B2	120.5 (2)
N1—B1—B2	120.40 (16)	F8—B8—B9	122.7 (2)
B5—B1—B4	60.53 (13)	B7—B8—B3	108.37 (16)
B5—B1—B6	60.43 (12)	B7—B8—B12	60.08 (14)
B5—B1—B2	108.85 (15)	B7—B8—B9	108.32 (18)
B4—B1—B6	109.03 (15)	B3—B8—B12	107.98 (17)
B4—B1—B2	109.34 (16)	B3—B8—B9	60.07 (14)
B3—B1—B5	109.28 (16)	B12—B8—B9	59.96 (15)
B3—B1—B4	60.73 (14)	B2—B8—B7	60.01 (14)
B3—B1—B6	108.75 (15)	B2—B8—B3	60.29 (14)
B3—B1—B2	60.58 (14)	B2—B8—B12	107.98 (17)
B2—B1—B6	59.93 (12)	B2—B8—B9	108.32 (16)
F5—B5—B1	122.70 (17)	F12—B12—B7	121.77 (19)
F5—B5—B11	121.44 (17)	F12—B12—B11	121.59 (18)
F5—B5—B4	122.07 (17)	F12—B12—B8	122.15 (18)
F5—B5—B6	121.61 (17)	F12—B12—B10	121.38 (19)
F5—B5—B10	121.63 (17)	F12—B12—B9	121.48 (19)
B1—B5—B11	107.20 (15)	B7—B12—B8	59.75 (15)
B1—B5—B4	59.80 (12)	B7—B12—B9	108.29 (17)
B1—B5—B6	60.03 (12)	B11—B12—B7	59.82 (13)
B1—B5—B10	107.51 (16)	B11—B12—B8	107.58 (17)
B4—B5—B11	107.98 (15)	B11—B12—B10	60.08 (13)
B4—B5—B6	108.13 (15)	B11—B12—B9	108.28 (16)
B6—B5—B11	59.44 (12)	B8—B12—B9	60.26 (16)
B10—B5—B11	60.00 (12)	B10—B12—B7	108.08 (16)
B10—B5—B4	60.11 (13)	B10—B12—B8	108.13 (16)
B10—B5—B6	107.69 (15)	B10—B12—B9	60.24 (15)
F7—B7—B11	121.85 (18)	F10—B10—B5	121.47 (19)
F7—B7—B6	121.44 (19)	F10—B10—B11	121.32 (19)
F7—B7—B8	121.95 (18)	F10—B10—B4	122.04 (18)
F7—B7—B12	122.20 (18)	F10—B10—B12	121.60 (18)
F7—B7—B2	121.48 (18)	F10—B10—B9	122.0 (2)
B11—B7—B8	108.03 (17)	B5—B10—B11	60.16 (13)
B11—B7—B12	59.96 (13)	B5—B10—B4	60.08 (13)
B11—B7—B2	107.76 (15)	B5—B10—B12	108.09 (16)
B6—B7—B11	59.77 (12)	B5—B10—B9	108.08 (17)
B6—B7—B8	107.85 (16)	B11—B10—B4	108.08 (16)
B6—B7—B12	107.77 (16)	B11—B10—B9	107.95 (17)
B6—B7—B2	59.88 (13)	B4—B10—B9	59.92 (14)
B8—B7—B12	60.17 (15)	B12—B10—B11	59.87 (13)
B8—B7—B2	59.97 (14)	B12—B10—B4	107.97 (17)
B2—B7—B12	108.01 (17)	B12—B10—B9	60.04 (14)
C5—C6—H6A	109.5	F2—B2—B1	122.02 (17)
C5—C6—H6B	109.5	F2—B2—B7	121.58 (17)
C5—C6—H6C	109.5	F2—B2—B3	121.96 (18)
H6A—C6—H6B	109.5	F2—B2—B6	121.21 (19)
H6A—C6—H6C	109.5	F2—B2—B8	122.18 (18)
H6B—C6—H6C	109.5	B1—B2—B7	107.82 (16)
C8—C9—H9A	109.5	B1—B2—B3	59.58 (13)
C8—C9—H9B	109.5	B1—B2—B6	60.20 (12)
C8—C9—H9C	109.5	B1—B2—B8	107.33 (17)
H9A—C9—H9B	109.5	B7—B2—B3	108.08 (17)
H9A—C9—H9C	109.5	B6—B2—B7	60.03 (13)
H9B—C9—H9C	109.5	B6—B2—B3	108.17 (16)
F11—B11—B5	120.67 (17)	B6—B2—B8	108.01 (16)
F11—B11—B7	121.67 (17)	B8—B2—B7	60.02 (14)
F11—B11—B6	120.32 (18)	B8—B2—B3	59.93 (14)
F11—B11—B12	123.00 (17)	F9—B9—B4	121.2 (2)
F11—B11—B10	122.20 (17)	F9—B9—B3	122.5 (2)
B7—B11—B5	108.36 (15)	F9—B9—B8	123.0 (2)
B7—B11—B12	60.22 (14)	F9—B9—B12	122.07 (19)
B7—B11—B10	108.37 (17)	F9—B9—B10	121.2 (2)
B6—B11—B5	60.28 (12)	B4—B9—B3	60.07 (14)
B6—B11—B7	60.11 (12)	B4—B9—B8	107.58 (18)
B6—B11—B12	108.24 (16)	B4—B9—B12	107.67 (18)
B6—B11—B10	108.23 (15)	B4—B9—B10	59.86 (14)
B12—B11—B5	107.98 (16)	B3—B9—B8	59.56 (15)
B12—B11—B10	60.05 (14)	B3—B9—B10	107.60 (17)
B10—B11—B5	59.84 (13)	B12—B9—B3	107.44 (19)
F4—B4—B1	121.42 (17)	B12—B9—B8	59.78 (15)
F4—B4—B5	120.51 (18)	B12—B9—B10	59.72 (14)
F4—B4—B3	122.59 (18)	B10—B9—B8	107.33 (18)
			
F11—B11—B6—F6	−0.9 (3)	B11—B10—B9—B3	63.0 (2)
F11—B11—B6—B1	147.93 (18)	B11—B10—B9—B8	0.3 (2)
F11—B11—B6—B5	110.3 (2)	B11—B10—B9—B12	−37.27 (16)
F11—B11—B6—B7	−111.4 (2)	B4—B1—B5—F5	110.9 (2)
F11—B11—B6—B2	−148.84 (18)	B4—B1—B5—B11	−101.17 (17)
F11—B11—B12—F12	−0.5 (3)	B4—B1—B5—B6	−138.70 (16)
F11—B11—B12—B7	110.5 (2)	B4—B1—B5—B10	−37.99 (15)
F11—B11—B12—B8	147.75 (19)	B4—B1—B3—F3	−110.5 (2)
F11—B11—B12—B10	−111.0 (2)	B4—B1—B3—B8	101.17 (19)
F11—B11—B12—B9	−148.6 (2)	B4—B1—B3—B2	138.77 (16)
F11—B11—B10—F10	1.5 (3)	B4—B1—B3—B9	37.55 (16)
F11—B11—B10—B5	−109.3 (2)	B4—B1—B6—F6	147.01 (18)
F11—B11—B10—B4	−146.95 (19)	B4—B1—B6—B5	37.43 (15)
F11—B11—B10—B12	112.3 (2)	B4—B1—B6—B7	−63.93 (19)
F11—B11—B10—B9	149.69 (19)	B4—B1—B6—B11	−0.6 (2)
F5—B5—B11—F11	0.9 (3)	B4—B1—B6—B2	−101.84 (18)
F5—B5—B11—B7	148.07 (19)	B4—B1—B2—F2	−148.4 (2)
F5—B5—B11—B6	110.6 (2)	B4—B1—B2—B7	63.4 (2)
F5—B5—B11—B12	−148.19 (18)	B4—B1—B2—B3	−37.54 (15)
F5—B5—B11—B10	−110.9 (2)	B4—B1—B2—B6	101.31 (17)
F5—B5—B4—F4	−1.0 (3)	B4—B1—B2—B8	0.1 (2)
F5—B5—B4—B1	−111.9 (2)	B4—B5—B11—F11	149.43 (18)
F5—B5—B4—B3	−148.52 (18)	B4—B5—B11—B7	−63.42 (19)
F5—B5—B4—B10	110.7 (2)	B4—B5—B11—B6	−100.86 (17)
F5—B5—B4—B9	148.08 (19)	B4—B5—B11—B12	0.3 (2)
F5—B5—B6—F6	−1.0 (3)	B4—B5—B11—B10	37.60 (15)
F5—B5—B6—B1	112.2 (2)	B4—B5—B6—F6	−150.00 (17)
F5—B5—B6—B7	−147.79 (18)	B4—B5—B6—B1	−36.88 (14)
F5—B5—B6—B11	−110.4 (2)	B4—B5—B6—B7	63.16 (19)
F5—B5—B6—B2	148.59 (18)	B4—B5—B6—B11	100.60 (17)
F5—B5—B10—F10	0.0 (3)	B4—B5—B6—B2	−0.5 (2)
F5—B5—B10—B11	110.6 (2)	B4—B5—B10—F10	111.4 (2)
F5—B5—B10—B4	−111.4 (2)	B4—B5—B10—B11	−137.98 (16)
F5—B5—B10—B12	147.83 (18)	B4—B5—B10—B12	−100.75 (18)
F5—B5—B10—B9	−148.66 (19)	B4—B5—B10—B9	−37.24 (16)
F6—B6—B2—F2	1.3 (3)	B4—B3—B8—F8	−149.7 (2)
F6—B6—B2—B1	112.8 (2)	B4—B3—B8—B7	63.4 (2)
F6—B6—B2—B7	−109.7 (2)	B4—B3—B8—B12	−0.2 (2)
F6—B6—B2—B3	149.52 (18)	B4—B3—B8—B2	100.69 (18)
F6—B6—B2—B8	−147.10 (18)	B4—B3—B8—B9	−37.58 (17)
F3—B3—B8—F8	0.1 (4)	B4—B3—B2—F2	147.77 (19)
F3—B3—B8—B7	−146.8 (2)	B4—B3—B2—B1	36.77 (15)
F3—B3—B8—B12	149.6 (2)	B4—B3—B2—B7	−63.7 (2)
F3—B3—B8—B2	−109.5 (2)	B4—B3—B2—B6	−0.2 (2)
F3—B3—B8—B9	112.2 (3)	B4—B3—B2—B8	−100.88 (18)
F3—B3—B2—F2	1.6 (3)	B4—B3—B9—F9	−110.0 (3)
F3—B3—B2—B1	−109.4 (2)	B4—B3—B9—B8	137.93 (18)
F3—B3—B2—B7	150.18 (19)	B4—B3—B9—B12	100.73 (19)
F3—B3—B2—B6	−146.30 (19)	B4—B3—B9—B10	37.79 (16)
F3—B3—B2—B8	113.0 (2)	B4—B10—B9—F9	110.4 (2)
F3—B3—B9—F9	−0.5 (4)	B4—B10—B9—B3	−37.88 (16)
F3—B3—B9—B4	109.5 (2)	B4—B10—B9—B8	−100.64 (18)
F3—B3—B9—B8	−112.5 (2)	B4—B10—B9—B12	−138.19 (18)
F3—B3—B9—B12	−149.7 (2)	B3—B1—B5—F5	148.45 (18)
F3—B3—B9—B10	147.3 (2)	B3—B1—B5—B11	−63.60 (19)
F4—B4—B3—F3	−0.6 (3)	B3—B1—B5—B4	37.57 (15)
F4—B4—B3—B1	−110.0 (2)	B3—B1—B5—B6	−101.14 (17)
F4—B4—B3—B8	149.99 (19)	B3—B1—B5—B10	−0.4 (2)
F4—B4—B3—B2	−146.79 (18)	B3—B1—B4—F4	111.9 (2)
F4—B4—B3—B9	112.2 (2)	B3—B1—B4—B5	−138.72 (16)
F4—B4—B10—F10	−1.3 (3)	B3—B1—B4—B10	−100.95 (18)
F4—B4—B10—B5	109.2 (2)	B3—B1—B4—B9	−37.57 (16)
F4—B4—B10—B11	146.82 (19)	B3—B1—B6—F6	−148.40 (19)
F4—B4—B10—B12	−149.87 (19)	B3—B1—B6—B5	102.02 (17)
F4—B4—B10—B9	−112.5 (2)	B3—B1—B6—B7	0.7 (2)
F4—B4—B9—F9	0.5 (4)	B3—B1—B6—B11	64.03 (19)
F4—B4—B9—B3	−111.5 (2)	B3—B1—B6—B2	−37.25 (16)
F4—B4—B9—B8	−148.8 (2)	B3—B1—B2—F2	−110.9 (2)
F4—B4—B9—B12	148.1 (2)	B3—B1—B2—B7	100.92 (19)
F4—B4—B9—B10	111.0 (2)	B3—B1—B2—B6	138.85 (17)
F10—B10—B9—F9	−0.8 (3)	B3—B1—B2—B8	37.64 (16)
F10—B10—B9—B4	−111.2 (2)	B3—B4—B10—F10	149.1 (2)
F10—B10—B9—B3	−149.03 (19)	B3—B4—B10—B5	−100.43 (17)
F10—B10—B9—B8	148.21 (19)	B3—B4—B10—B11	−62.8 (2)
F10—B10—B9—B12	110.7 (2)	B3—B4—B10—B12	0.5 (2)
F12—B12—B10—F10	−0.5 (3)	B3—B4—B10—B9	37.91 (17)
F12—B12—B10—B5	−148.3 (2)	B3—B4—B9—F9	112.1 (3)
F12—B12—B10—B11	−110.9 (2)	B3—B4—B9—B8	−37.30 (17)
F12—B12—B10—B4	148.19 (19)	B3—B4—B9—B12	−100.3 (2)
F12—B12—B10—B9	110.9 (2)	B3—B4—B9—B10	−137.52 (18)
F12—B12—B9—F9	−0.7 (4)	B3—B8—B12—F12	−148.0 (2)
F12—B12—B9—B4	−147.9 (2)	B3—B8—B12—B7	101.27 (19)
F12—B12—B9—B3	148.7 (2)	B3—B8—B12—B11	64.0 (2)
F12—B12—B9—B8	111.6 (2)	B3—B8—B12—B10	0.5 (2)
F12—B12—B9—B10	−110.7 (2)	B3—B8—B12—B9	−37.46 (17)
F7—B7—B11—F11	−1.2 (3)	B3—B8—B2—F2	111.0 (2)
F7—B7—B11—B5	−147.9 (2)	B3—B8—B2—B1	−37.48 (15)
F7—B7—B11—B6	−110.4 (2)	B3—B8—B2—B7	−138.44 (17)
F7—B7—B11—B12	111.4 (2)	B3—B8—B2—B6	−100.99 (17)
F7—B7—B11—B10	148.7 (2)	B3—B8—B9—F9	−111.1 (3)
F7—B7—B6—F6	1.6 (3)	B3—B8—B9—B4	37.52 (16)
F7—B7—B6—B1	−148.48 (19)	B3—B8—B9—B12	138.12 (18)
F7—B7—B6—B5	148.58 (19)	B3—B8—B9—B10	100.60 (18)
F7—B7—B6—B11	111.1 (2)	B6—B1—B5—F5	−110.4 (2)
F7—B7—B6—B2	−110.7 (2)	B6—B1—B5—B11	37.53 (15)
F7—B7—B8—F8	0.7 (3)	B6—B1—B5—B4	138.70 (16)
F7—B7—B8—B3	147.9 (2)	B6—B1—B5—B10	100.72 (16)
F7—B7—B8—B12	−111.5 (2)	B6—B1—B4—F4	−146.77 (19)
F7—B7—B8—B2	110.5 (2)	B6—B1—B4—B5	−37.38 (14)
F7—B7—B8—B9	−148.5 (2)	B6—B1—B4—B3	101.33 (17)
F7—B7—B12—F12	−0.2 (3)	B6—B1—B4—B10	0.4 (2)
F7—B7—B12—B11	−110.9 (2)	B6—B1—B4—B9	63.8 (2)
F7—B7—B12—B8	111.1 (2)	B6—B1—B3—F3	147.7 (2)
F7—B7—B12—B10	−148.0 (2)	B6—B1—B3—B4	−101.80 (17)
F7—B7—B12—B9	148.2 (2)	B6—B1—B3—B8	−0.6 (2)
F7—B7—B2—F2	0.3 (3)	B6—B1—B3—B2	36.97 (15)
F7—B7—B2—B1	148.6 (2)	B6—B1—B3—B9	−64.3 (2)
F7—B7—B2—B3	−148.4 (2)	B6—B1—B2—F2	110.3 (2)
F7—B7—B2—B6	110.6 (2)	B6—B1—B2—B7	−37.93 (16)
F7—B7—B2—B8	−111.3 (2)	B6—B1—B2—B3	−138.85 (17)
O1—C2—C1—Se1	−2.6 (2)	B6—B1—B2—B8	−101.21 (17)
O3—C8—C7—Se1	1.0 (2)	B6—B5—B11—F11	−109.7 (2)
F8—B8—B12—F12	1.3 (4)	B6—B5—B11—B7	37.43 (15)
F8—B8—B12—B7	−109.4 (3)	B6—B5—B11—B12	101.17 (17)
F8—B8—B12—B11	−146.8 (2)	B6—B5—B11—B10	138.45 (17)
F8—B8—B12—B10	149.8 (2)	B6—B5—B4—F4	147.86 (18)
F8—B8—B12—B9	111.8 (3)	B6—B5—B4—B1	36.98 (14)
F8—B8—B2—F2	−0.4 (3)	B6—B5—B4—B3	0.4 (2)
F8—B8—B2—B1	−148.9 (2)	B6—B5—B4—B10	−100.41 (16)
F8—B8—B2—B7	110.1 (2)	B6—B5—B4—B9	−63.0 (2)
F8—B8—B2—B3	−111.4 (2)	B6—B5—B10—F10	−147.41 (19)
F8—B8—B2—B6	147.6 (2)	B6—B5—B10—B11	−36.83 (15)
F8—B8—B9—F9	−0.6 (4)	B6—B5—B10—B4	101.15 (17)
F8—B8—B9—B4	148.0 (2)	B6—B5—B10—B12	0.4 (2)
F8—B8—B9—B3	110.5 (2)	B6—B5—B10—B9	63.9 (2)
F8—B8—B9—B12	−111.4 (2)	B6—B7—B11—F11	109.2 (2)
F8—B8—B9—B10	−148.9 (2)	B6—B7—B11—B5	−37.50 (15)
O2—C5—C4—Se1	−9.3 (2)	B6—B7—B11—B12	−138.15 (17)
N1—B1—B5—F5	1.9 (3)	B6—B7—B11—B10	−100.92 (17)
N1—B1—B5—B11	149.81 (17)	B6—B7—B8—F8	−147.2 (2)
N1—B1—B5—B4	−109.0 (2)	B6—B7—B8—B3	0.0 (2)
N1—B1—B5—B6	112.3 (2)	B6—B7—B8—B12	100.66 (17)
N1—B1—B5—B10	−147.01 (17)	B6—B7—B8—B2	−37.34 (16)
N1—B1—B4—F4	3.6 (3)	B6—B7—B8—B9	63.7 (2)
N1—B1—B4—B5	113.0 (2)	B6—B7—B12—F12	147.91 (19)
N1—B1—B4—B3	−108.3 (2)	B6—B7—B12—B11	37.25 (15)
N1—B1—B4—B10	150.76 (17)	B6—B7—B12—B8	−100.79 (17)
N1—B1—B4—B9	−145.85 (18)	B6—B7—B12—B10	0.1 (2)
N1—B1—B3—F3	0.1 (3)	B6—B7—B12—B9	−63.7 (2)
N1—B1—B3—B4	110.61 (19)	B6—B7—B2—F2	−110.3 (2)
N1—B1—B3—B8	−148.22 (18)	B6—B7—B2—B1	38.01 (16)
N1—B1—B3—B2	−110.62 (19)	B6—B7—B2—B3	100.97 (17)
N1—B1—B3—B9	148.16 (17)	B6—B7—B2—B8	138.13 (17)
N1—B1—B6—F6	−2.4 (3)	B6—B11—B12—F12	−148.5 (2)
N1—B1—B6—B5	−112.0 (2)	B6—B11—B12—B7	−37.52 (15)
N1—B1—B6—B7	146.64 (17)	B6—B11—B12—B8	−0.2 (2)
N1—B1—B6—B11	−149.98 (17)	B6—B11—B12—B10	100.97 (17)
N1—B1—B6—B2	108.7 (2)	B6—B11—B12—B9	63.5 (2)
N1—B1—B2—F2	−2.7 (3)	B6—B11—B10—F10	148.15 (19)
N1—B1—B2—B7	−150.88 (18)	B6—B11—B10—B5	37.33 (15)
N1—B1—B2—B3	108.2 (2)	B6—B11—B10—B4	−0.3 (2)
N1—B1—B2—B6	−112.9 (2)	B6—B11—B10—B12	−100.99 (17)
N1—B1—B2—B8	145.84 (18)	B6—B11—B10—B9	−63.6 (2)
C3—C2—C1—Se1	178.11 (17)	B8—B7—B11—F11	−150.19 (19)
B1—B5—B11—F11	−147.51 (18)	B8—B7—B11—B5	63.1 (2)
B1—B5—B11—B7	−0.4 (2)	B8—B7—B11—B6	100.57 (17)
B1—B5—B11—B6	−37.80 (15)	B8—B7—B11—B12	−37.58 (16)
B1—B5—B11—B12	63.4 (2)	B8—B7—B11—B10	−0.3 (2)
B1—B5—B11—B10	100.66 (17)	B8—B7—B6—F6	149.62 (19)
B1—B5—B4—F4	110.9 (2)	B8—B7—B6—B1	−0.4 (2)
B1—B5—B4—B3	−36.63 (15)	B8—B7—B6—B5	−63.4 (2)
B1—B5—B4—B10	−137.39 (16)	B8—B7—B6—B11	−100.87 (19)
B1—B5—B4—B9	−100.02 (18)	B8—B7—B6—B2	37.38 (17)
B1—B5—B6—F6	−113.1 (2)	B8—B7—B12—F12	−111.3 (2)
B1—B5—B6—B7	100.05 (17)	B8—B7—B12—B11	138.04 (16)
B1—B5—B6—B11	137.48 (16)	B8—B7—B12—B10	100.87 (18)
B1—B5—B6—B2	36.42 (14)	B8—B7—B12—B9	37.10 (17)
B1—B5—B10—F10	149.29 (18)	B8—B7—B2—F2	111.5 (2)
B1—B5—B10—B11	−100.14 (16)	B8—B7—B2—B1	−100.12 (19)
B1—B5—B10—B4	37.85 (15)	B8—B7—B2—B3	−37.15 (16)
B1—B5—B10—B12	−62.9 (2)	B8—B7—B2—B6	−138.13 (17)
B1—B5—B10—B9	0.6 (2)	B8—B3—B2—F2	−111.3 (2)
B1—B4—B3—F3	109.4 (2)	B8—B3—B2—B1	137.65 (17)
B1—B4—B3—B8	−100.03 (18)	B8—B3—B2—B7	37.19 (16)
B1—B4—B3—B2	−36.81 (15)	B8—B3—B2—B6	100.71 (18)
B1—B4—B3—B9	−137.81 (17)	B8—B3—B9—F9	112.1 (3)
B1—B4—B10—F10	−148.2 (2)	B8—B3—B9—B4	−137.93 (18)
B1—B4—B10—B5	−37.70 (15)	B8—B3—B9—B12	−37.20 (16)
B1—B4—B10—B11	−0.1 (2)	B8—B3—B9—B10	−100.15 (19)
B1—B4—B10—B12	63.2 (2)	B8—B12—B10—F10	−149.3 (2)
B1—B4—B10—B9	100.63 (19)	B8—B12—B10—B5	62.9 (2)
B1—B4—B9—F9	149.4 (2)	B8—B12—B10—B11	100.27 (19)
B1—B4—B9—B3	37.35 (16)	B8—B12—B10—B4	−0.6 (2)
B1—B4—B9—B8	0.0 (2)	B8—B12—B10—B9	−37.96 (18)
B1—B4—B9—B12	−63.0 (2)	B8—B12—B9—F9	−112.3 (3)
B1—B4—B9—B10	−100.17 (17)	B8—B12—B9—B4	100.4 (2)
B1—B3—B8—F8	147.2 (2)	B8—B12—B9—B3	37.11 (17)
B1—B3—B8—B7	0.4 (2)	B8—B12—B9—B10	137.68 (18)
B1—B3—B8—B12	−63.2 (2)	B12—B7—B11—F11	−112.6 (2)
B1—B3—B8—B2	37.62 (16)	B12—B7—B11—B5	100.65 (17)
B1—B3—B8—B9	−100.65 (19)	B12—B7—B11—B6	138.15 (17)
B1—B3—B2—F2	111.0 (2)	B12—B7—B11—B10	37.24 (15)
B1—B3—B2—B7	−100.46 (17)	B12—B7—B6—F6	−146.85 (18)
B1—B3—B2—B6	−36.94 (15)	B12—B7—B6—B1	63.1 (2)
B1—B3—B2—B8	−137.65 (17)	B12—B7—B6—B5	0.2 (2)
B1—B3—B9—F9	−147.5 (2)	B12—B7—B6—B11	−37.34 (15)
B1—B3—B9—B4	−37.47 (15)	B12—B7—B6—B2	100.92 (18)
B1—B3—B9—B8	100.46 (18)	B12—B7—B8—F8	112.2 (2)
B1—B3—B9—B12	63.3 (2)	B12—B7—B8—B3	−100.62 (19)
B1—B3—B9—B10	0.3 (2)	B12—B7—B8—B2	−138.00 (17)
B1—B6—B2—F2	−111.6 (2)	B12—B7—B8—B9	−36.97 (16)
B1—B6—B2—B7	137.50 (17)	B12—B7—B2—F2	149.1 (2)
B1—B6—B2—B3	36.67 (15)	B12—B7—B2—B1	−62.5 (2)
B1—B6—B2—B8	100.06 (18)	B12—B7—B2—B3	0.5 (2)
B5—B1—B4—F4	−109.4 (2)	B12—B7—B2—B6	−100.51 (17)
B5—B1—B4—B3	138.72 (16)	B12—B7—B2—B8	37.62 (16)
B5—B1—B4—B10	37.77 (15)	B12—B11—B6—F6	148.10 (18)
B5—B1—B4—B9	101.15 (17)	B12—B11—B6—B1	−63.08 (19)
B5—B1—B3—F3	−148.0 (2)	B12—B11—B6—B5	−100.73 (17)
B5—B1—B3—B4	−37.48 (15)	B12—B11—B6—B7	37.57 (16)
B5—B1—B3—B8	63.7 (2)	B12—B11—B6—B2	0.2 (2)
B5—B1—B3—B2	101.28 (16)	B12—B11—B10—F10	−110.9 (2)
B5—B1—B3—B9	0.1 (2)	B12—B11—B10—B5	138.32 (17)
B5—B1—B6—F6	109.6 (2)	B12—B11—B10—B4	100.71 (18)
B5—B1—B6—B7	−101.36 (17)	B12—B11—B10—B9	37.35 (16)
B5—B1—B6—B11	−37.98 (15)	B12—B8—B2—F2	−148.1 (2)
B5—B1—B6—B2	−139.27 (17)	B12—B8—B2—B1	63.4 (2)
B5—B1—B2—F2	147.1 (2)	B12—B8—B2—B7	−37.57 (16)
B5—B1—B2—B7	−1.1 (2)	B12—B8—B2—B3	100.87 (19)
B5—B1—B2—B3	−102.00 (17)	B12—B8—B2—B6	−0.1 (2)
B5—B1—B2—B6	36.85 (15)	B12—B8—B9—F9	110.7 (2)
B5—B1—B2—B8	−64.4 (2)	B12—B8—B9—B4	−100.60 (19)
B5—B11—B6—F6	−111.2 (2)	B12—B8—B9—B3	−138.12 (18)
B5—B11—B6—B1	37.65 (14)	B12—B8—B9—B10	−37.52 (16)
B5—B11—B6—B7	138.29 (16)	B12—B10—B9—F9	−111.4 (2)
B5—B11—B6—B2	100.88 (16)	B12—B10—B9—B4	138.19 (18)
B5—B11—B12—F12	147.8 (2)	B12—B10—B9—B3	100.3 (2)
B5—B11—B12—B7	−101.30 (17)	B12—B10—B9—B8	37.55 (16)
B5—B11—B12—B8	−64.0 (2)	B10—B5—B11—F11	111.8 (2)
B5—B11—B12—B10	37.19 (15)	B10—B5—B11—B7	−101.02 (18)
B5—B11—B12—B9	−0.3 (2)	B10—B5—B11—B6	−138.45 (17)
B5—B11—B10—F10	110.8 (2)	B10—B5—B11—B12	−37.29 (16)
B5—B11—B10—B4	−37.61 (15)	B10—B5—B4—F4	−111.7 (2)
B5—B11—B10—B12	−138.32 (17)	B10—B5—B4—B1	137.39 (16)
B5—B11—B10—B9	−100.97 (17)	B10—B5—B4—B3	100.76 (17)
B5—B4—B3—F3	146.05 (19)	B10—B5—B4—B9	37.37 (17)
B5—B4—B3—B1	36.69 (14)	B10—B5—B6—F6	146.48 (18)
B5—B4—B3—B8	−63.3 (2)	B10—B5—B6—B1	−100.40 (17)
B5—B4—B3—B2	−0.1 (2)	B10—B5—B6—B7	−0.4 (2)
B5—B4—B3—B9	−101.12 (17)	B10—B5—B6—B11	37.08 (15)
B5—B4—B10—F10	−110.5 (2)	B10—B5—B6—B2	−63.98 (19)
B5—B4—B10—B11	37.64 (15)	B10—B11—B6—F6	−148.32 (18)
B5—B4—B10—B12	100.95 (18)	B10—B11—B6—B1	0.5 (2)
B5—B4—B10—B9	138.34 (18)	B10—B11—B6—B5	−37.14 (15)
B5—B4—B9—F9	−147.6 (2)	B10—B11—B6—B7	101.15 (18)
B5—B4—B9—B3	100.33 (18)	B10—B11—B6—B2	63.74 (19)
B5—B4—B9—B8	63.0 (2)	B10—B11—B12—F12	110.6 (2)
B5—B4—B9—B12	0.0 (2)	B10—B11—B12—B7	−138.49 (17)
B5—B4—B9—B10	−37.19 (16)	B10—B11—B12—B8	−101.20 (18)
B5—B6—B2—F2	−147.83 (17)	B10—B11—B12—B9	−37.52 (17)
B5—B6—B2—B1	−36.28 (15)	B10—B4—B3—F3	−150.83 (19)
B5—B6—B2—B7	101.22 (17)	B10—B4—B3—B1	99.81 (17)
B5—B6—B2—B3	0.4 (2)	B10—B4—B3—B8	−0.2 (2)
B5—B6—B2—B8	63.8 (2)	B10—B4—B3—B2	63.0 (2)
B5—B10—B9—F9	147.71 (19)	B10—B4—B3—B9	−38.00 (16)
B5—B10—B9—B4	37.31 (16)	B10—B4—B9—F9	−110.4 (3)
B5—B10—B9—B3	−0.6 (2)	B10—B4—B9—B3	137.52 (18)
B5—B10—B9—B8	−63.3 (2)	B10—B4—B9—B8	100.22 (19)
B5—B10—B9—B12	−100.88 (18)	B10—B4—B9—B12	37.17 (17)
B7—B11—B6—F6	110.5 (2)	B10—B12—B9—F9	110.0 (3)
B7—B11—B6—B1	−100.64 (17)	B10—B12—B9—B4	−37.24 (17)
B7—B11—B6—B5	−138.29 (16)	B10—B12—B9—B3	−100.57 (19)
B7—B11—B6—B2	−37.41 (15)	B10—B12—B9—B8	−137.68 (18)
B7—B11—B12—F12	−110.9 (2)	B2—B1—B5—F5	−147.05 (19)
B7—B11—B12—B8	37.29 (16)	B2—B1—B5—B11	0.9 (2)
B7—B11—B12—B10	138.49 (17)	B2—B1—B5—B4	102.07 (18)
B7—B11—B12—B9	100.97 (19)	B2—B1—B5—B6	−36.64 (15)
B7—B11—B10—F10	−148.17 (19)	B2—B1—B5—B10	64.1 (2)
B7—B11—B10—B5	101.01 (16)	B2—B1—B4—F4	149.37 (19)
B7—B11—B10—B4	63.4 (2)	B2—B1—B4—B5	−101.24 (16)
B7—B11—B10—B12	−37.31 (15)	B2—B1—B4—B3	37.48 (15)
B7—B11—B10—B9	0.0 (2)	B2—B1—B4—B10	−63.5 (2)
B7—B6—B2—F2	110.9 (2)	B2—B1—B4—B9	−0.1 (2)
B7—B6—B2—B1	−137.50 (17)	B2—B1—B3—F3	110.7 (2)
B7—B6—B2—B3	−100.83 (18)	B2—B1—B3—B4	−138.77 (16)
B7—B6—B2—B8	−37.44 (16)	B2—B1—B3—B8	−37.60 (17)
B7—B8—B12—F12	110.7 (2)	B2—B1—B3—B9	−101.22 (18)
B7—B8—B12—B11	−37.32 (15)	B2—B1—B6—F6	−111.2 (2)
B7—B8—B12—B10	−100.78 (18)	B2—B1—B6—B5	139.27 (17)
B7—B8—B12—B9	−138.73 (17)	B2—B1—B6—B7	37.91 (16)
B7—B8—B2—F2	−110.6 (2)	B2—B1—B6—B11	101.28 (17)
B7—B8—B2—B1	100.96 (17)	B2—B7—B11—F11	146.45 (19)
B7—B8—B2—B3	138.44 (17)	B2—B7—B11—B5	−0.3 (2)
B7—B8—B2—B6	37.45 (15)	B2—B7—B11—B6	37.21 (16)
B7—B8—B9—F9	147.8 (2)	B2—B7—B11—B12	−100.95 (19)
B7—B8—B9—B4	−63.6 (2)	B2—B7—B11—B10	−63.7 (2)
B7—B8—B9—B3	−101.09 (18)	B2—B7—B6—F6	112.2 (2)
B7—B8—B9—B12	37.03 (16)	B2—B7—B6—B1	−37.81 (16)
B7—B8—B9—B10	−0.5 (2)	B2—B7—B6—B5	−100.75 (17)
B7—B12—B10—F10	147.5 (2)	B2—B7—B6—B11	−138.26 (17)
B7—B12—B10—B5	−0.3 (2)	B2—B7—B8—F8	−109.8 (3)
B7—B12—B10—B11	37.06 (16)	B2—B7—B8—B3	37.38 (16)
B7—B12—B10—B4	−63.8 (2)	B2—B7—B8—B12	138.00 (17)
B7—B12—B10—B9	−101.17 (19)	B2—B7—B8—B9	101.03 (18)
B7—B12—B9—F9	−149.2 (2)	B2—B7—B12—F12	−148.83 (19)
B7—B12—B9—B4	63.6 (2)	B2—B7—B12—B11	100.51 (17)
B7—B12—B9—B3	0.2 (2)	B2—B7—B12—B8	−37.53 (15)
B7—B12—B9—B8	−36.87 (16)	B2—B7—B12—B10	63.3 (2)
B7—B12—B9—B10	100.81 (18)	B2—B7—B12—B9	−0.4 (2)
C6—C5—C4—Se1	171.83 (17)	B2—B3—B8—F8	109.6 (3)
C9—C8—C7—Se1	−178.89 (16)	B2—B3—B8—B7	−37.26 (17)
B11—B5—B4—F4	−149.29 (18)	B2—B3—B8—B12	−100.86 (19)
B11—B5—B4—B1	99.84 (16)	B2—B3—B8—B9	−138.27 (18)
B11—B5—B4—B3	63.21 (19)	B2—B3—B9—F9	149.3 (2)
B11—B5—B4—B10	−37.55 (15)	B2—B3—B9—B4	−100.68 (17)
B11—B5—B4—B9	−0.2 (2)	B2—B3—B9—B8	37.26 (16)
B11—B5—B6—F6	109.4 (2)	B2—B3—B9—B12	0.1 (2)
B11—B5—B6—B1	−137.48 (16)	B2—B3—B9—B10	−62.9 (2)
B11—B5—B6—B7	−37.43 (15)	B2—B8—B12—F12	148.2 (2)
B11—B5—B6—B2	−101.06 (17)	B2—B8—B12—B7	37.54 (16)
B11—B5—B10—F10	−110.6 (2)	B2—B8—B12—B11	0.2 (2)
B11—B5—B10—B4	137.98 (16)	B2—B8—B12—B10	−63.2 (2)
B11—B5—B10—B12	37.23 (15)	B2—B8—B12—B9	−101.20 (18)
B11—B5—B10—B9	100.75 (18)	B2—B8—B9—F9	−148.7 (2)
B11—B7—B6—F6	−109.5 (2)	B2—B8—B9—B4	0.0 (2)
B11—B7—B6—B1	100.45 (16)	B2—B8—B9—B3	−37.52 (16)
B11—B7—B6—B5	37.51 (15)	B2—B8—B9—B12	100.60 (18)
B11—B7—B6—B2	138.26 (17)	B2—B8—B9—B10	63.1 (2)
B11—B7—B8—F8	149.7 (2)	B9—B4—B3—F3	−112.8 (2)
B11—B7—B8—B3	−63.1 (2)	B9—B4—B3—B1	137.81 (17)
B11—B7—B8—B12	37.49 (15)	B9—B4—B3—B8	37.78 (17)
B11—B7—B8—B2	−100.51 (17)	B9—B4—B3—B2	101.00 (18)
B11—B7—B8—B9	0.5 (2)	B9—B4—B10—F10	111.1 (2)
B11—B7—B12—F12	110.7 (2)	B9—B4—B10—B5	−138.34 (18)
B11—B7—B12—B8	−138.04 (16)	B9—B4—B10—B11	−100.69 (19)
B11—B7—B12—B10	−37.18 (16)	B9—B4—B10—B12	−37.39 (17)
B11—B7—B12—B9	−100.94 (18)	B9—B3—B8—F8	−112.2 (3)
B11—B7—B2—F2	−147.5 (2)	B9—B3—B8—B7	101.0 (2)
B11—B7—B2—B1	0.8 (2)	B9—B3—B8—B12	37.41 (18)
B11—B7—B2—B3	63.8 (2)	B9—B3—B8—B2	138.27 (18)
B11—B7—B2—B6	−37.16 (15)	B9—B3—B2—F2	−148.9 (2)
B11—B7—B2—B8	100.96 (19)	B9—B3—B2—B1	100.14 (18)
B11—B6—B2—F2	148.37 (17)	B9—B3—B2—B7	−0.3 (2)
B11—B6—B2—B1	−100.08 (16)	B9—B3—B2—B6	63.2 (2)
B11—B6—B2—B7	37.42 (15)	B9—B3—B2—B8	−37.51 (17)
B11—B6—B2—B3	−63.41 (19)	B9—B8—B12—F12	−110.6 (2)
B11—B6—B2—B8	0.0 (2)	B9—B8—B12—B7	138.73 (17)
B11—B12—B10—F10	110.4 (2)	B9—B8—B12—B11	101.41 (18)
B11—B12—B10—B5	−37.36 (15)	B9—B8—B12—B10	37.95 (17)
B11—B12—B10—B4	−100.89 (17)	B9—B8—B2—F2	148.4 (2)
B11—B12—B10—B9	−138.23 (18)	B9—B8—B2—B1	−0.1 (2)
B11—B12—B9—F9	147.5 (2)	B9—B8—B2—B7	−101.02 (19)
B11—B12—B9—B4	0.2 (2)	B9—B8—B2—B3	37.42 (17)
B11—B12—B9—B3	−63.1 (2)	B9—B8—B2—B6	−63.6 (2)
B11—B12—B9—B8	−100.23 (19)	B9—B12—B10—F10	−111.4 (2)
B11—B12—B9—B10	37.45 (16)	B9—B12—B10—B5	100.87 (19)
B11—B10—B9—F9	−148.7 (2)	B9—B12—B10—B11	138.23 (18)
B11—B10—B9—B4	100.92 (17)	B9—B12—B10—B4	37.34 (17)

### Hydrogen-bond geometry (Å, º)

**Table:**

*D*—H···*A*	*D*—H	H···*A*	*D*···*A*	*D*—H···*A*
N1—H···F8^i^	0.91	2.13	2.871 (2)	138
N1—H···O2^ii^	0.91	2.20	2.865 (2)	129
N1—H*A*···O1^iii^	0.91	1.94	2.836 (2)	168
N1—H*B*···O3^iv^	0.91	1.94	2.841 (2)	171

Symmetry codes: (i) *x*+1/2, −*y*+3/2, −*z*+1; (ii) *x*, −*y*+3/2, *z*−1/2; (iii) *x*+1/2, *y*, −*z*+3/2; (iv) −*x*+1, *y*+1/2, −*z*+3/2.
